# Editorial: Building tomorrow's biomedical workforce: advancing scholarship, innovation and systemic change

**DOI:** 10.3389/frma.2025.1652438

**Published:** 2025-07-11

**Authors:** Adriana Bankston, Rebekah L. Layton, Audra Van Wart

**Affiliations:** ^1^AAAS/ASGCT Congressional Policy Fellow, U.S. House of Representatives, Washington, DC, United States; ^2^Department of Microbiology and Immunology, University of North Carolina at Chapel Hill School of Medicine, Chapel Hill, NC, United States; ^3^Office of Biomedical Science Training and Research, University of North Carolina at Chapel Hill School of Medicine, Chapel Hill, NC, United States; ^4^Department of Medical Science, Division of Biology and Medicine, Brown University, Providence, RI, United States

**Keywords:** biomedical, workforce, education, training, research, careers, professional development, culture change

Sustaining innovation and excellence in the U.S. biomedical research enterprise depends on cultivating a skilled workforce prepared to address future challenges and excel across a growing array of professional roles. Today's biomedical scientists face a variety of challenges across the career trajectory, including limited exposure to the full breadth of career paths, extended training timelines, heightened uncertainty around career prospects, hypercompetition, the growing instability of research funding, and exclusion of scientists from historically underrepresented groups. These issues are compounded by structural systems that under-acknowledge contributions to team science, innovation, and mentoring, relative to metrics like publications and grants (Alberts et al., 2014; Pickett et al., 2015; National Academy of Sciences, Engineering, and Medicine, 2014, 2018; National Academies of Sciences, Engineering, and Medicine, 2019; Council of Graduate Schools, 2025; Okahana, 2019). Addressing these challenges is essential to maintaining U.S. competitiveness and creating pathways that attract, retain, and prepare a highly talented and diverse pool of scientists contributing to the STEM workforce and broader economy (National Institutes of Health, 2012). Our Research Topic, *Building Tomorrow's Biomedical Workforce: Evaluation of How Evidence-Based Training Programs Align Skill Development and Career Awareness with a Broad Array of Professions*, tackles these and other challenges, in order to develop the researchers of tomorrow.

Authors featured in this Research Topic explore approaches toward effective training and education of biomedical scientists, and their preparation for STEM and research-related roles, covering the continuum of academic career development from undergraduate and graduate education through postdoctoral training and early career faculty roles. In this context, our Research Topic highlights contributions of a growing community of dedicated education researchers who are impacting pressing issues in biomedical education, training and workforce development through rigorous, evidence-based, data-driven research. By expanding the growing knowledge base in this field (Van Wart et al., 2020), we aim to support academic leaders and national changemakers in implementing transformative programs, curricula, and policies that foster the development of a robust STEM and biomedical workforce.

The articles selected for inclusion in our Research Topic clustered into three major thematic pillars: (1) career transitions, academic job market, and career outcomes; (2) career and professional skill development in programs and curricula; and (3) policies, perspectives, and theories to advance systemic change to build a thriving, diverse research training ecosystem. The first pillar examines professional development with respect to workforce trends. The second pillar combines professional development topics (including wellness and experiential learning) with program design. The third pillar highlights processes and approaches that encourage diverse perspectives and foster supportive biomedical research communities.

These pillars align with priorities of organizations that have helped support this type of work for decades and which regularly facilitate discussions among the biomedical education and training community, such as Rescuing Biomedical Research (Rescuing Biomedical Research, 2025), the National Postdoctoral Association (National Postdoctoral Association, 2025), the Graduate Career Consortium (Graduate Career Consortium, 2025), and the Association of American Medical Colleges (AAMC) Group on Research Education and Training (Association of American Medical Colleges, 2025). Federal science agencies, including the National Institutes of Health (NIH) and the National Science Foundation (NSF), have historically recognized the importance of workforce development through funding initiatives like NIH Broadening Experiences in Scientific Training (NIH BEST) (National Institutes of Health, 2013); A Science of Science Approach to Analyzing and Innovating the Biomedical Research Enterprise (SoS:BIO, formerly SCISIPBIO; National Institute of General Medical Sciences, 2025; National Science Foundation, 2019; Zuk, 2019); Maximizing Opportunities for Scientific and Academic Independent Careers (National Institutes of Health, 2024; Grants.gov, 2025); and Innovative Programs to Enhance Research Training (National Institutes of Health, 2025). These initiatives have supported the researcher pipeline, facilitated testing of new training approaches, and promoted standardized data collection efforts in biomedical education research.

Additionally, programs from Burroughs Wellcome Fund [Career Grants; (Burroughs Wellcome Fund, 2025); (BWF)] and Howard Hughes Medical Institute (Howard Hughes Medical Foundation, 2025) have further supported research in education, training and career development to enable biomedical research talent development through partnerships with academic institutions. Collectively, the coordinated efforts of entities from various sectors, including academic institutions, hospitals, research institutes, federal agencies, and non-profits, have helped expand research efforts on biomedical workforce development, and have grown the community of educators and scholars committed to advancing STEM training and career readiness.

While we hope our Research Topic will further the growth of the field and inspire institutional reforms, concepts discussed in the articles have taken on a new dimension given the shift in federal policies that occurred shortly after publication of many of these pieces, creating uncertainty about the future of STEM training. As of this publication date, academic investigators nationwide are experiencing delays in federal grant reviews, and recent actions by the administration have led to significant science funding cuts at agencies supporting research and STEM workforce development, including the National Institutes of Health (Kozlov, 2025) and National Science Foundation (Boodman, 2025). Such funding cuts have not only impacted the pursuit of research innovations at U.S. institutions, but also their educational and public service missions by limiting capacity to support trainees in undergraduate, graduate, and postdoctoral research pursuits, and by delaying, canceling, or eliminating federally funded training programs designed to build a strong biomedical workforce. Still, institutions must carry forward and remain grounded in their values. Our Research Topic offers insights that may help institutions fulfill their missions.

Publications in the first pillar focus on ***Career Transitions, Career Outcomes, and the*** ***Academic Job Market***, exploring critical aspects of career transitions and outcomes for graduate students and postdoctoral scholars, and highlighting strategies to better align academic training with long-term career success. Collins et al. synthesize existing PhD career taxonomies and visualization tools to guide institutions in publishing career outcomes, thereby helping trainees make informed decisions about where to pursue their education and training. For PhD graduates interested in academic careers, Cresiski et al. address the disconnect between postdoctoral training and workforce development in higher education by offering PROMISE Academy Fellows structured exposure to diverse institution types, including tours, workshops, and speaking engagements. Flynn et al. investigate academic job market trends in biological sciences, identifying key factors that influence faculty job offers (including application volume, gender, and ethnicity or race), and noting that completing multiple postdoctoral appointments does not necessarily improve academic hiring outcomes. *Together, these studies offer practical insights for improving transparency and effectiveness in career preparation across the academic pipeline*.

Publications in the second pillar focus on ***Career and Professional Skill Development in*** ***Programs and Curricula***, highlighting the need for student-centered training that provide professional skills and competencies needed to succeed in transitioning from academic training into the workforce. Focusing on transferable professional skills, Nguyen introduces a cohort-based design thinking program for postdoctoral scholars that fosters professional skill development, community building, and belonging—key factors for career preparation. Similarly, Salm and McKinney develop the Academy for Transferable Management Skills (ATMS), enabling graduate students and postdoctoral scholars to apply project management frameworks within their research, strengthening readiness for careers across multiple sectors. Finally, Ragland et al. describe Duke's BioCoRE program, which prepares graduate students for non-academic careers through soft skills training in communication, conflict resolution, time management, and job market readiness.

Several publications in the second pillar focus on psychosocial skills and wellness. For example, Safer et al. propose integrating psychosocial skill development (such as motivation, self-regulation, and social-relational skills) across the doctoral training experience via curricula and workshops. Responding to heightened stress and isolation during the COVID-19 pandemic, Han et al. pilot a resiliency program that improved trainees' adaptability, self-efficacy, and mental wellbeing. Shifting to topics of independence and career readiness, Singh et al. explore ways to promote independence in early-career researchers through multi-institutional workshops that revealed barriers to and solutions for developing the independence of thought needed to advance in academic careers. Likewise, Neely et al. evaluate the long-running OPTIONS program, demonstrating how experiential learning and reflection can enhance career readiness across doctoral programs. Identifying a need to establish and more structurally measure PhD level progress within career and professional development programs, Davidson et al. introduce customized qualifying exams to include student-centered, career-aligned competencies, thereby improving their individual relevance without increasing burden. *Together, these studies offer innovative, scalable strategies for enhancing the professional and career preparedness of biomedical trainees*.

Publications in the third pillar focus on ***Policies, Perspectives, and Theories to Advance*** ***Systemic Change to Build a Thriving Research Training Ecosystem***, aiming to promote the use of theory-driven, evidence-based practices in decision-making across higher education. Brandt and Robinson emphasize the scholarly potential of program directors for collecting and disseminating the data needed to effect institutional change. They advocate for practitioner-scholar models in which academic leaders actively engage in publishing on training innovations, and provide examples of contributions to institutional improvement and influence national policies. Continuing with the theme of structural change, several articles identify opportunities for promoting inclusive and equitable training experiences. Baldwin-SoRelle and McDonald focus on the experiences of LGBTQ+ graduate students, highlighting safety and inclusion challenges in academia and offering recommendations for improved classroom and lab environments. Montgomery and Black argue for multidisciplinary theoretical frameworks to guide systemic change in academic settings, introducing the “groundskeeping” framework as a strategy for driving equity-focused institutional reform. They offer a step-by-step roadmap and national examples to guide institutions and scientific organizations in assessing current practices for moving toward systemic change, dismantling structural barriers, and fostering sustainable, inclusive environments. With the potential to inform and improve national funding models, Torres et al. perform a literature review that examines structural inequities in STEM research funding for trainee-caregivers, especially women, and propose actionable policies to promote inclusion, improve transparency, and support more equitable institutional funding outcomes. Focusing on the role of mentorship, Packard et al. develop a systems-level approach to STEM mentoring through an ecosystems framework, which emphasizes shifting from isolated efforts to coordinated, institution-wide strategies supporting workforce diversification, collaboration, and long-term mentoring effectiveness. Finally, two articles examine the impact of academic recruitment approaches for promoting and sustaining a diverse STEM workforce. Arruda et al. discuss long-term outcomes of institutional recruitment and student support strategies, which show success in retaining a diverse biomedical workforce and providing rare longitudinal data on academic persistence by race, ethnicity, and gender. Finally, Cho et al. present successful holistic admissions and recruitment practices in academia, demonstrating how intentional design can result in improved retention of students with diverse identities and perspectives in research careers. *Together, these studies offer actionable frameworks and data-driven insights for building more inclusive, equitable, and effective research training environments*.

The articles published in our Research Topic collectively argue for a critical need to develop structured and improved support mechanisms for academic and non-academic career transitions, professional skill development, systemic policy changes, and inclusivity within biomedical training. This Research Topic underscores the need for evidence-based institutional training programs that enhance career awareness, skill-building, and mentoring for trainees, while also addressing psychosocial factors and structural barriers influencing trainee career trajectories. Additionally, research results presented by our authors emphasize the positive impact which inclusive policies, community-building initiatives, and systemic interventions and their implementation, can have in fostering a diverse, equitable, and well-trained biomedical workforce prepared to address societal challenges.

The biomedical research ecosystem nationwide faces growing challenges in implementing innovative strategies, and building upon long-established practices that strengthen much needed skills, foster supportive training environments, as well as recruit and retain trainees in scientific fields (Mervis, 2025). This culture change needs a strong focus on ensuring that trainees from multiple backgrounds can join and contribute to the scientific community (Asai, 2020). We also need to broaden the pool of investigators receiving federal research funding and those who review and manage these awards, in order to create a robust STEM workforce at multiple levels (Bernard et al., 2021). Recently, federal funding for several long-standing programs aimed at advancing a more inclusive scientific research pipeline has been delayed or terminated, leaving institutions with the difficult task of having to fill the gaps in research training that were once supported by these awards. These programs previously supported by federal grant mechanisms include training grants that facilitate the development of cohort-based support networks, faculty-based research awards and associated diversity supplements, as well as trainee fellowships. If intentional efforts are not made to create an environment where trainees from every background can thrive, we will lose top talent from our research laboratories and institutions. Coupled with broader cuts to federal research funding, many institutions are now admitting fewer graduate students and postdoctoral researchers, which means that a reduced number of talented scientists are entering and remaining in research careers long-term (Molteni et al., 2025). For trainees that enter the research pipeline, there is an immediate need prepare them for a wide range of careers that may include non-academic routes in the United States at a time when science funding is scarce and STEM talent may be lost to other countries (Patel, 2025). Over the long term, the loss of research talent could compound to negatively impact American innovation and productivity, and reduce the overall resiliency of the biomedical research enterprise.

As the research enterprise evolves, academic training programs will need to adapt in order to meet shifting demands of today's dynamic federal policy environment. **This evolution requires a sustained national commitment to researching and advancing evidence-based practices in training and career development, in order to support a robust biomedical workforce**. By building on past successes and integrating proven strategies, we can avoid repeating known pitfalls and advance scalable, high-impact solutions grounded in strong federal research investments. Institutions can partner with funding agencies and private funders to test biomedical research training innovations, support rigorous programming, enhance opportunities, and collect national data important for understanding workforce development. Our Research Topic supports the broader goal of building and sustaining a strong biomedical research workforce by showcasing innovative programs and elevating diverse perspectives in academia that foster creativity, resilience, and progress in scientific careers.

A key aim of our Research Topic has been increasing the visibility of scholarship in academic research training and education as an emerging discipline, which may strengthen efforts to sustain the biomedical workforce across multiple career paths. We hope that our Research Topic will catalyze similar future efforts from scholars and practitioners by providing data-driven insights and promoting the dissemination and implementation of effective educational practices and frameworks to prepare the workforce for tomorrow's challenges. In today's turbulent federal policy environment, the systemic changes outlined in several articles from our Research Topic are essential for helping the biomedical research enterprise respond effectively to challenges and build a resilient, future-ready workforce. This Research Topic is intended to be accessible and valuable to a wide range of audiences, while providing insights for academic leaders and federal decision-makers whose influence can positively shape the biomedical research ecosystem through effective implementation.
